# Lack of association between methylenetetrahydrofolate reductase C677T polymorphism, HPV infection and cervical intraepithelial neoplasia in Brazilian women

**DOI:** 10.1186/s12881-019-0831-x

**Published:** 2019-06-06

**Authors:** Nayara Nascimento Toledo Silva, Adriano de Paula Sabino, Alexandre Tafuri, Angélica Alves Lima

**Affiliations:** 1 Programa de Pós-Graduação em Ciências Farmacêuticas, Departamento de Análises Clínicas, Universidade Federal de Ouro Preto, Ouro Preto, Minas Gerais 35400-000 Brazil; 2 Departamento de Análises Clínicas e Toxicológicas, Universidade Federal de Minas Gerais, President Antônio Carlos Avenue, 6627, Belo Horizonte, Minas Gerais 31270-901 Brazil; 3 Laboratório Tafuri, São Paulo Street, 893, Belo Horizonte, Minas Gerais 30170-131 Brazil

**Keywords:** HPV, Cervical Cancer, SNP, MTHFR C677T polymorphism, Folate metabolism

## Abstract

**Background:**

Cervical cancer has high prevalence and mortality rates in worldwide female population. Persistent infection by high-risk Human Papillomavirus (hr-HPV) is the main cause of this cancer. However, many environmental, genetical, and epigenetical cofactors can modulate viral infection and cervical carcinogenesis. Methylenetetrahydrofolate reductase (MTHFR) C677T polymorphism is a genetic factor that has been associated with many pathologies, including cancer. Nevertheless, studies with cervical cancer presented controversial results, and varied according to ethnicity. Thus, the aim of this study was to determine association between MTHFR C677T polymorphism, Human Papillomavirus (HPV) infection and cervical cancer.

**Methods:**

A case-control study was performed with 150 histological cervical samples. Case group were divided in Cervical Intraepithelial Neoplasia (CIN) grade I (*n* = 30), CIN II (*n* = 30), CIN III (*n* = 30), and Squamous Cervical Carcinoma (SCC) (*n* = 30). Control group was composed by 30 samples without lesion, presenting cervicitis. HPV detection was performed by conventional Polymerase Chain Reaction (PCR) with SPF primers set, and by real-time PCR specific for HPV 16 and hr-HPV. MTHFR C677T polymorphism was analyzed by PCR followed by Restriction Fragment Length Polymorphism (RFLP).

**Results:**

Frequency of MTHFR CC genotype was 72.7% (*n* = 109), CT 23.3% (*n* = 35) and TT 4.0% (*n* = 6). Polymorphic T allele frequency was 15.7%. No statistically significant association was observed between MTHFR C677T polymorphism and presence of pre-neoplastic or neoplastic cervical lesions. Similar frequencies of T allele was observed in control (23.3%) and cases (13.3%) groups (*p* = 0.174). In addition, there was no statistically significant association between MTHFR C677T polymorphism and viral infection, even considering hr-HPV or HPV 16 positivity.

**Conclusion:**

MTHFR C677T polymorphism was not associated with cervical cancer and HPV infection.

## Background

Cervical cancer is the fourth most common cancer in women worldwide, with 529,000 new cases, and 275,000 deaths each year. Most cases occur in developing countries. On the other hand, lower incidence rates of this tumor occur in developed countries, where programs for prevention, screening and treatment are well established [1]. In Brazil, cervical cancer is the third type of tumor that most often affects female population, with an annual mortality rate of 5000 women. Furthermore, 16,370 new cases were estimated in Brazil for 2018 [2].

Persistent infection with high-risk Human Papillomavirus (hr-HPV) is the main cause of cervical cancer, and 99.7% of cases are associated with the virus [3]. However, only 10% of women presenting HPV infection will develop precancerous lesions, and less than 1% of these abnormalities will progress, leading to cervical tumor [4]. Thus, presence of other factors in cervical cancer development is necessary in addition to HPV infection. Currently, several genetics, epigenetics, and environmental factors had been studied [5, 6].

Methylenetetrahydrofolate Reductase (MTHFR) is an enzyme that plays a central role in folate metabolism, important in cell replication and gene expression [7]. C677T polymorphism was identified by Frost and colleagues in 1995, and leads to the exchange of cytosine (C) to thymine (T) at codon 222, resulting in reduced enzymatic activity of MTHFR [8]. This Single Nucleotide Polymorphism (SNP) is the most common MTHFR genetic variant found in population, wherein frequency of the TT polymorphic genotype may vary according to ethnicity [8, 9]. In US population, frequency of polymorphic allele is approximately 35%, while Asian and Caucasian populations present 12 to 15% rates of TT individuals, and greater than 50% for CT genotype. Moreover, the incidence of polymorphic genotype is less than 1% among American Afro-descendants [10].

MTHFR C677T polymorphism has been associated with development of cardiovascular diseases, defects in the neural tube formation, psychiatric disorders and various cancers [9]. The presence of polymorphic T allele was considered a protective factor for colorectal cancer, acute lymphoblastic leukemia in adults, and some leukemias and lymphomas in children. However, MTHFR C677T polymorphism has been identified as a risk factor for breast, endometrium, esophagus, stomach, pancreas, and bladder cancer [11, 12]. Few studies have been conducted to evaluate the frequency of MTHFR C677T polymorphism and its association with cervical cancer. Besides, results were controversial and inconclusive, varying according to country and ethnicity [13, 14].

Thus, the aim of this study was to determine association between MTHFR C677T polymorphism, HPV infection and cervical cancer in Brazilian women.

## Methods

### Samples selection

Samples were collected between 2006 and 2011, from Brazilian women in Minas Gerais State, Brazil, a population with highly mixed ethnicities. Rating Histology of Richart was used to classify the samples [15]. Tafuri Laboratory kindly provided these blocks of cervical biopsies. Thirty samples presenting Squamous Cervical Cancer (SCC) were provided to perform the study. Therefore, we composed the other groups [Control, Cervical Intraepithelial Neoplasia grade I (CIN I), CIN II, and CIN III], with the same number of samples. These were chosen randomly from database of Laboratory Tafuri, pairing the age according to the patients from SCC group.

**Fig. 1:**
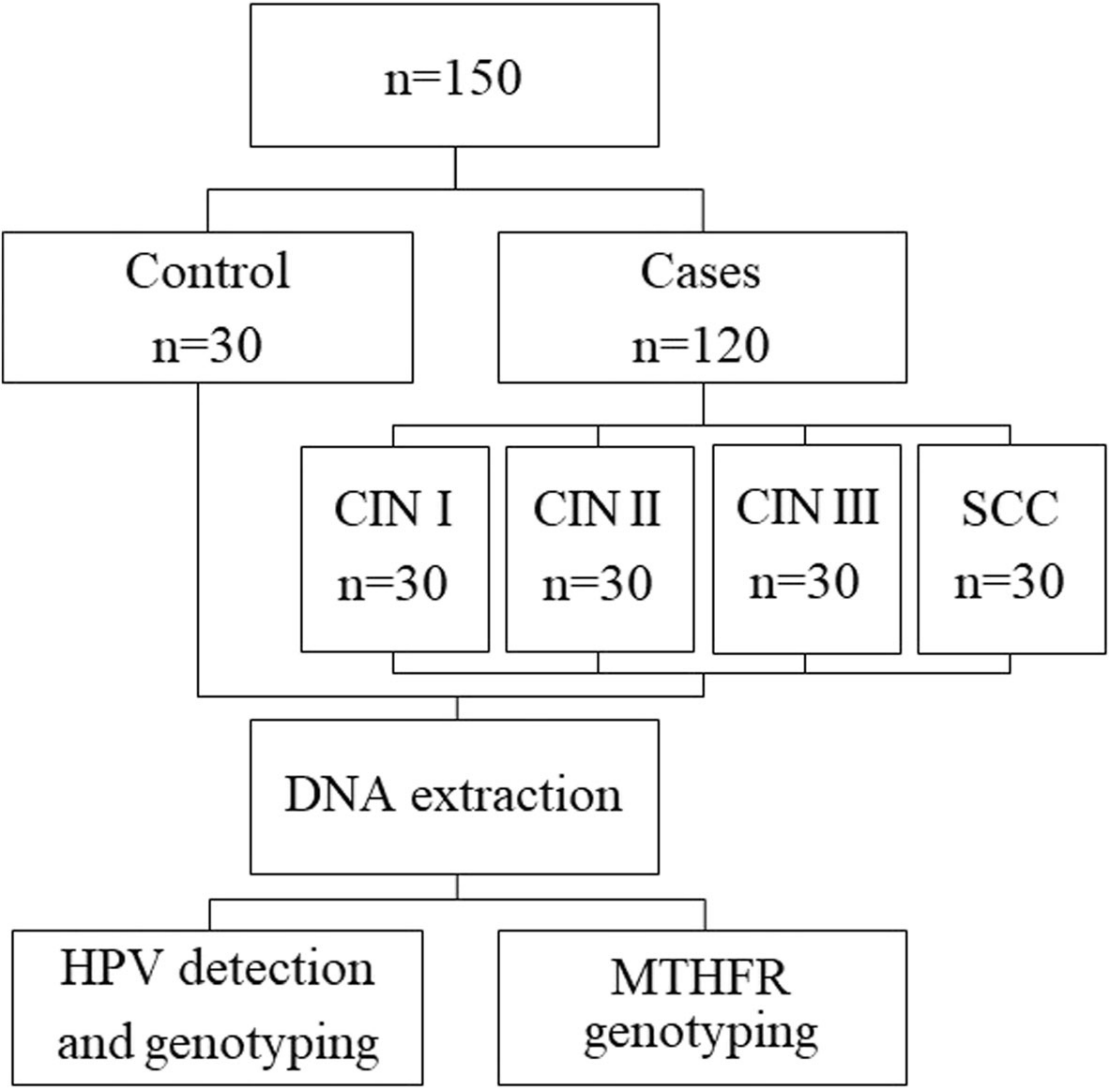
Study flow diagram. CIN: Cervical Intraepithelial Neoplasia; HPV: Human Papillomavirus; MTHFR: Methylenetetrahydrofolate Reductase

Thus, case group was composed of 120 histological samples divided into CIN I (*n* = 30), CIN II (*n* = 30), CIN III (*n* = 30), and SCC (*n* = 30). Thirty samples without cervical dysplasia was considered the control group (Fig. 1).

This study was approved by Research Ethics Committee of the Federal University of Ouro Preto.

### Preparation and DNA extraction

DNA extraction was performed using three histological sections of 10 μm of each biopsy block (Fig. 1). Firstly, the paraffin was removed with xylene and absolute ethanol. DNA extraction was performed with QIAamp® DNA FFPE Tissue Kit (Qiagen, Hilden, Germany). Concentration and purity of extracted DNA were determined by NanoDrop 2000® spectrophotometer (Thermo Scientific, Waltham, Massachusetts, United States of America).

### HPV detection

**Fig. 2:**
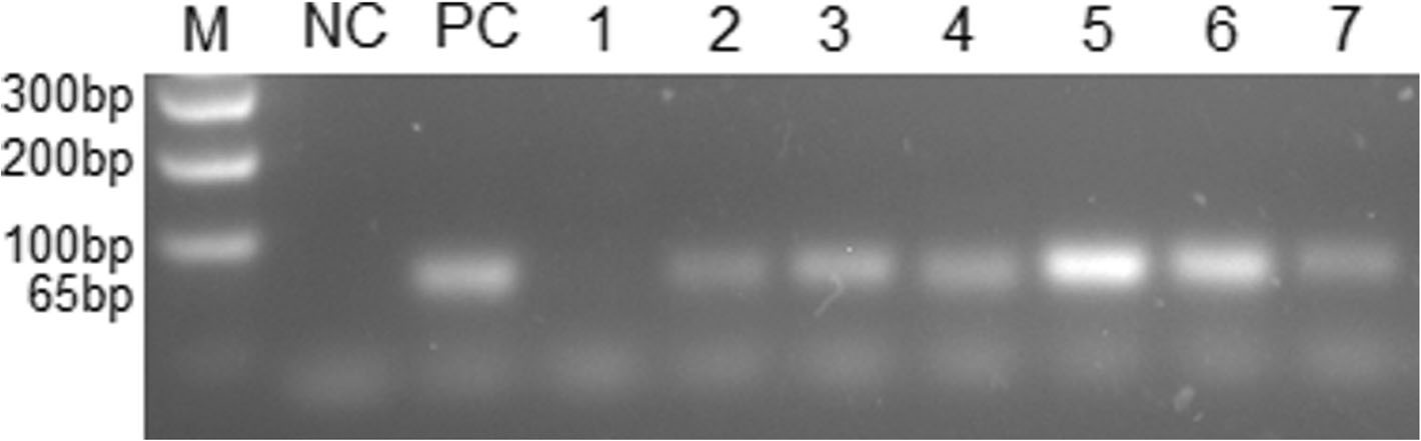
HPV detection by PCR with SPF primers set. M: 100 bp DNA ladders; NP: negative control; PC: Positive control; 1: HPV negative sample; 2–7: HPV positive samples

Generic HPV detection was performed by PCR with SPF primers set [16], that detect all HPV types (Fig. 1). Protocol and PCR conditions were presented in Tables 1 and 2, respectively. PCR products were identified using agarose gel electrophoresis stained with GelRed® (Biotium, Fremont, California, United States of America). Presence of HPV infection was confirmed with fragment amplification of 65 bp (Fig. 2).

**Table 1:** PCR protocol for HPV detection with SPF primers set

Reagents	Protocol
PCR Master Mix®^a^	12.5 μl
Primers^b^ (10 pmol/μl)	0.5 μl (each)
Sample (DNA)	25 ng
Final volume	25 μl

^a^0.2 mM each deoxyribonucleotide (dNTP), 1.5 mM MgCl_2_ and 1.0 unit of Taq DNA (Promega, Madison, Wisconsin, United States of America)

^b^Nucleotide sequences (5′-3′): SPF 1A: GCiCAGGGiCACAATAATGG; SPF 1B: GCiCAGGGiCATAACAATGG; SPF 1C: GCiCAGGGiCATAATAATGG; SPF 1D: GCiCAAGGiCATAATAATGG; SPF 2B: GTiGTATCiACAACAGTAACAAA; SPF 2D: GTiGTATCiACTACAGTAACAAA

**Table 2:** PCR condition for HPV detection with SPF primers set

Number of cycles	Temperature (°C)	Time (minutes)	Stage
1	94	1	Denaturation
40	94	1	Denaturation
	45	1	Annealing
	72	1	Extension
1	72	5	Final Extension

HPV genotyping was performed by real-time PCR with Bio Gene HPV Alto Risco PCR® (Bioclin, Belo Horizonte, Minas Gerais, Brazil). This assay is based on qualitative detection of hr-HPV (16, 18, 31, 33, 35, 39, 45, 51, 52, 56, 58, 59, 66 e 68), according to recommendations of manufacturer, and distinguish the presence of HPV 16.

### MTHFR C677T polymorphism analysis

MTHFR C677T polymorphism was analyzed by PCR followed by Restriction Fragment Length Polymorphism (RFLP) (Fig. 1), as described by Silva et al. [17]. Fragments generated by RFLP were analyzed by capillary electrophoresis performed on the QIAxcel Advanced System® (Qiagen, Hilden, Germany). CC individuals presented one fragment of 198 bp, heterozygotes (CT) presented two fragments of 198 bp and 175 bp, and polymorphic individuals (TT) presented one fragment of 175 bp.

### Statistical analysis

Data were tabulated by Microsoft Office Excel® (Microsoft, Redmond, Washington, United States of Amercia,) and analyzed by Statistical Package for the Social Sciences® 17.0 (International Business Machines, New York, United States of America).

Descriptive statistics were performed to evaluate the frequency of MTHFR genotypes and HPV infection. Allelic frequency was calculated by Genepop software [18]. Frequency distribution and X^2^ test following Monte-Carlo simulation were used to compare the groups.

To evaluate the effect of age on the association between MTHFR C677T polymorphism and cervical carcinogenesis, samples were divided into two age groups using 30 years old as the cut off. After this age, oncogenic HPV infection is more persistent, and women are more likely to have pre-invasive lesion.

Hardy-Weinberg Equilibrium (HWE) of MTHFR genotypes frequencies was calculated by Hardy-Weinberg equilibrium calculator including analysis for ascertainment bias [19].

*p* values < 0.05 were considered as evidence of a significant association in all tests and 95% confidence interval was calculated when appropriate.

## Results

Age range of women varied from 15 to 82 years, and mean and standard deviation of case and control groups were respectively 39.1 ± 13.5 and 40,5 ± 15.9 years.

Genotypes frequencies of MTHFR C677T polymorphism were 72.7% (*n* = 109), 23.3% (*n* = 35) and 4.0% (*n* = 6) of CC, CT and TT, respectively. Frequency of polymorphic T allele was 15.7%. Distribution of genotypes was found under HWE (X^2^ = 0.14, *p* = .0708).

**Fig. 3:**
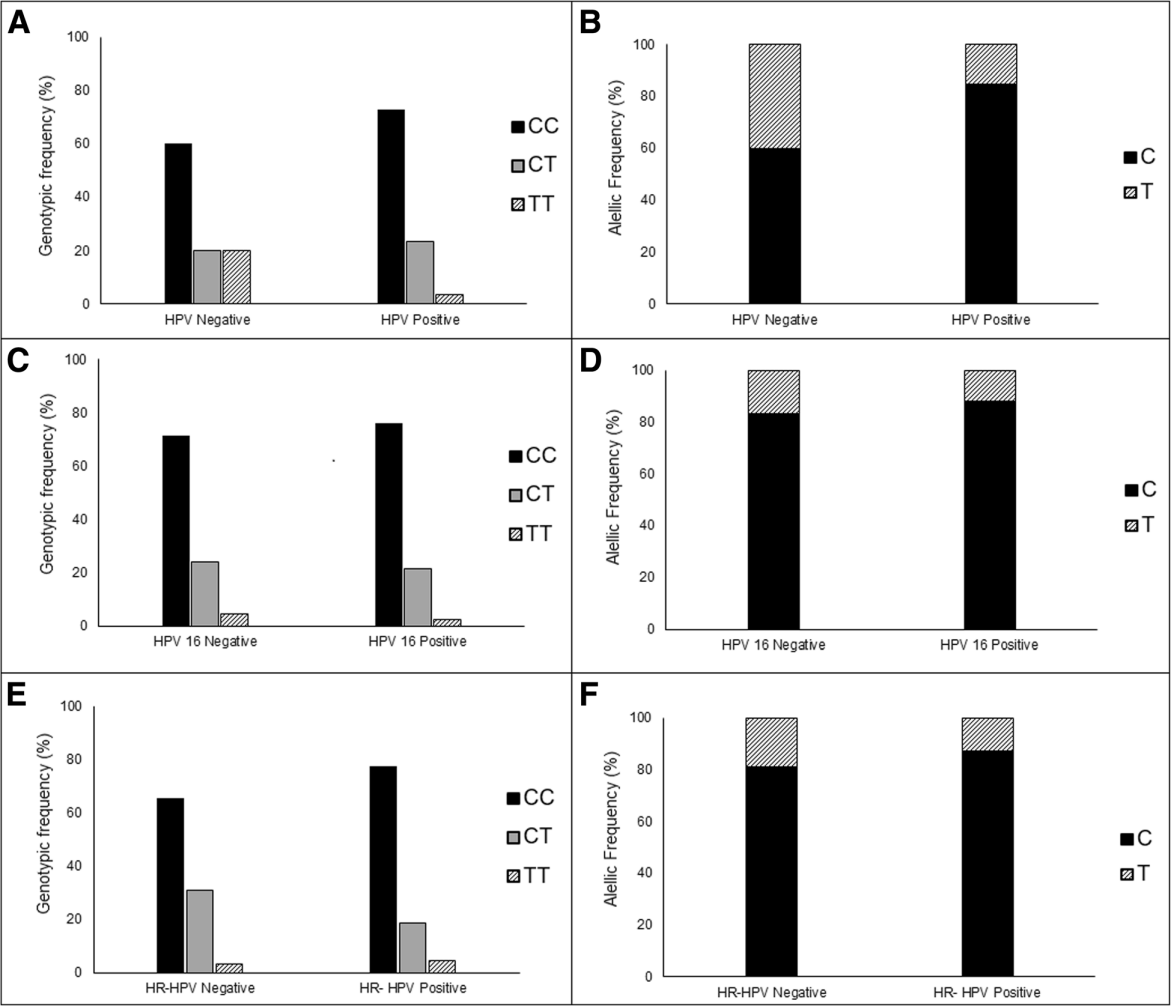
MTHFR genotypic and allelic frequency in relation to HPV infection. Frequency of CC, CT and TT genotypes (**a**, **c**, **e**), and C and T alleles (**b**, **d**, **f**) according HPV infection (**a**, **b**), HPV 16 infection (**c**, **d**), and hr-HPV infection (**e**, **f**)

HPV infection was found in 96.7% (*n* = 145) of samples. Most of samples presented hr-HPV infection (61.3%, *n* = 92). Beside this, HPV 16 positivity was 28.0% (*n* = 42). However, no statistically significant difference was observed between distribution of MTHFR genotypes or alleles and HPV infection status (Fig. 3).

**Fig. 4:**
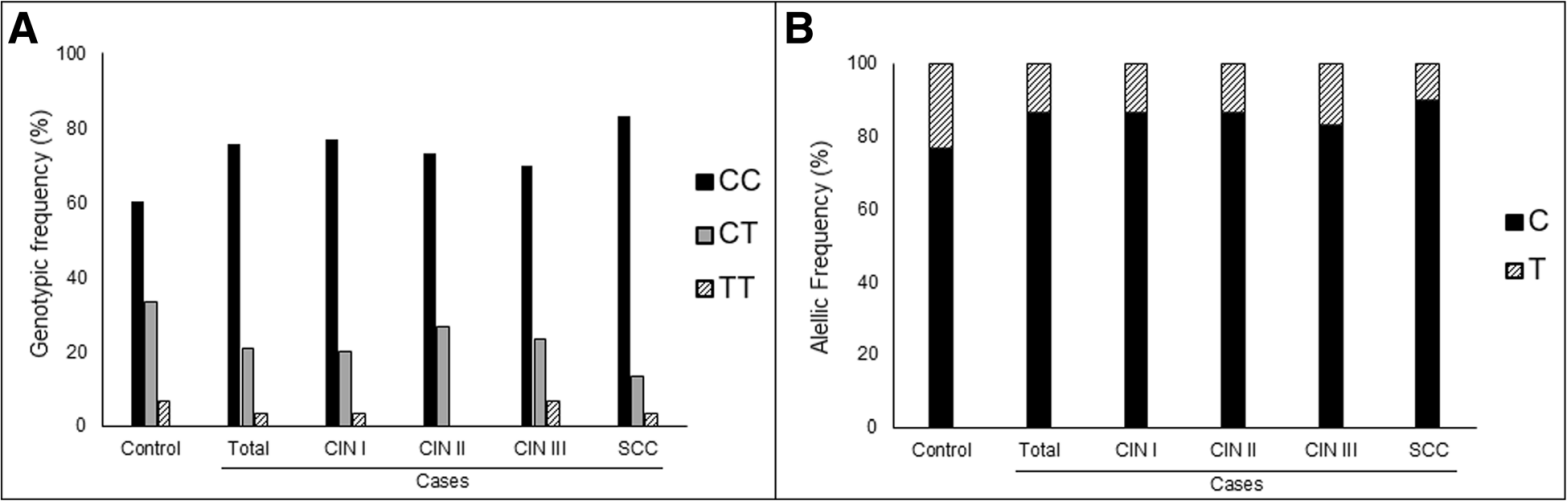
MTHFR genotypic and allelic frequency according to cervical abnormalities. Frequency of CC, CT and TT genotypes (**a**), and C and T alleles (**b**) in Control and Case groups (Total, CIN I, CIN II, CIN III, and SCC)

There was no statistically significant association between distribution of MTHFR genotypes (*p* = 0.212) and alleles (*p* = 0.174), and presence of CIN or SCC (case group) (Fig. 4). Similarly, no association was observed according to lesions degree in uterine cervix (genotypes: *p* = 0.600; alleles: *p* = 0.669) (Fig. 4). Same results were obtained analyzing only HPV, hr-HPV or HPV 16 positive samples (results not showed).

**Fig. 5:**
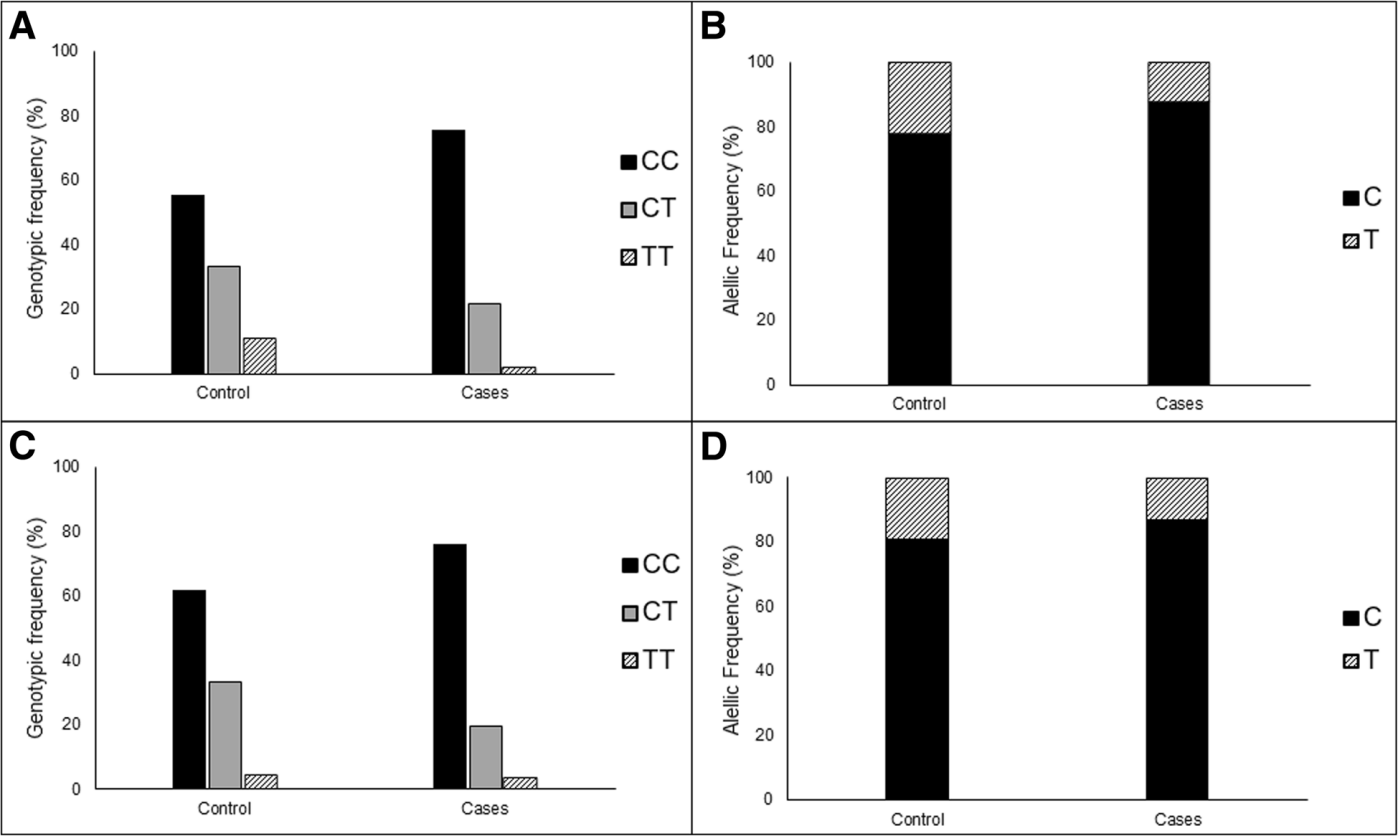
Association between MTHFR C677T polymorphism and cervical lesions according to the age group. Frequencies of CC, CT and TT genotypes (**a**, **c**), and C and T alleles (**b**, **d**) among women with age ≤ 30 years old (**a**, **b**) and > 30 years old (**c**, **d**) in Control and Case groups. Note: Three samples were excluded due to lack of age data

To evaluate the effect of age on the association between MTHFR C677T polymorphism and cervical carcinogenesis, samples were divided into two age groups: ≤30 and > 30 years. However, results did not show significant differences (*p* > 0.05) between distribution of genotypic and allelic frequencies and presence of cervical abnormalities according age group (Fig. 5).

## Discussion

MTHFR C677T polymorphism may modify the susceptibility to carcinogenesis by modulating the availability of 5,10-methylenetetrahydrofolate at different sites of folate metabolism. This coenzymatic form of folate plays a central role in folate metabolism, because 5,10-methylenetetrahydrofolate can be (a) directly transferred to deoxyuridine monophosphate (dUMP) in thymidylate synthesis; (b) oxidized to 10-formyltetrahydrofolate for de novo synthesis of purines; or (c) reduced to 5-methyltetrahydrofolate by MTHFR for methionine synthesis. Thus, reduction of enzymatic activity of MTHFR caused by C677T polymorphism may result in a lower synthesis rate of 5-methyltetrahydrofolate, leading to an increase availability of 5,10-methylenetetrahydrofolate for nucleotide production, which is essential for DNA synthesis and repair. In contrast, a lower proportion of 5-methyltetrahydrofolate is available for the methylation pathway, affecting genic expression [20, 21].

However, although MTHFR C677T polymorphism may be linked to carcinogenesis by altering nucleotide synthesis, and DNA methylation pattern, in this study it was not possible to observe association between the presence of polymorphic genotype or allele, and cervical cancer. Other authors have also found no relationship between polymorphism in MTHFR gene and cervical carcinogenesis [22–31]. However, studies performed with women from United States and Mexico indicated the presence of the polymorphism as a protective factor for cervical cancer development [21, 32]. Furthermore, a recent study in Iran also showed the presence of this SNP as a protective factor for cervical cancer [33].

In Brazil, only one study had been performed to evaluate the relationship between MTHFR C677T polymorphism and cervical cancer [34]. The authors analyzed data of 950 women attended in Brazilian Cancer Control Institute and Pérola Byington Hospital, both located in São Paulo, from 2003 to 2005. Unlike our result, Tomita et al. [34] observed the presence of MTHFR C677T polymorphism as a risk factor for cervical cancer. This difference may be occurred because Brazil is a country of great territorial extension, in which a highly mixed population is observed, presenting differences not only between its geographic regions but also between the states that compose them. Meta-analyses observed that C677T MTHFR polymorphism may be associated with cervical carcinogenesis in a different way according to ethnicity. This SNP was considered risk factor for cervical cancer among Asiatic women, but protection factor for Caucasian [13, 14, 35, 36].

No association between HPV infection and C677T polymorphism in MTHFR gene were observed. We did not find other studies performed with Brazilian women, and analyzing association between MTHFR C677T polymorphism and viral infection. However, similar results were obtained with Indian population [24, 37]. On the other hand, studies conducted in United States and Romania indicated the presence of MTHFR C677T polymorphism as a risk factor for HPV infection [38, 39]. In contrast, Hajiesmaeil and colleagues [33] observed CT genotype as a protective factor for viral infection between Iranian women.

Age is a cofactor that could also be considered. Development of cervical cancer begins with hr-HPV infection. If infection persists for 1 or 2 years, cervical abnormalities may occur. Only about 10% viral infection progress, leading to low-grade lesions. Approximately 2% of infected women have high-grade cervical lesions after years of persistent infection. High-grade abnormalities usually expand laterally around the transformation zone of cervical tissue, leading to invasive squamous cell carcinoma. Thus, there are more likely age groups for each stage of cervical cancer, with the highest prevalence of HPV infection occurring during adolescence and early adulthood, while the highest incidence of high-grade lesions is seen in women aged between 25 and 35 years. Carcinoma occurs predominantly between 45 and 60 years of age. Moreover, oncogenic HPV infection is more persistent in women aged 30 years or older, age group that women are more likely to have pre-invasive lesions [4, 40].

However, we did not observe significant difference in distribution of MTHFR genotype between cases and controls in both group ages evaluated (≤30 and > 30 years old). On the other hand, Sull and colleagues [41] concluded that the presence of the polymorphic genotype increases the risk of developing cervical cancer in Korean women younger than 40 years.

Some meta-analysis found that MTHFR C677T polymorphism is associated with cervical carcinogenesis differently according to ethnic group, acting as a risk factor for Asian, and as a protective factor for Caucasian [13, 14]. However, our study was performed with Brazilian women, a population composed by many mixed ethnicities. Thus, was not possible analyzing the role of polymorphism in cervical cancer development considering different ethnicities separately.

Besides, cervical carcinogenesis is a multifactorial disease, although HPV infection is the main factor. Consequently, lack of information about other factors must be considered, like sexual behavior, multiparity, smoking and drinking habits, presence of other sexually transmitted diseases, and other genetic factors [6].

Moreover, absence of data on serum folate of participants should be taken into account, since effect of MTHFR C677T polymorphism on HPV infection and development of cervical cancer can be modified by levels of folic acid [27, 42]. MTHFR polymorphism associated with low folate levels leads to changes in DNA methylation pattern of host cells as well as in HPV genome, which causes changes in gene expression that could lead to infection persistence. In addition, DNA integrity may be compromised, increasing chance of integration of HPV genetic material into host DNA, a key process for progression of infection to cancer [43].

## Conclusions

In summary, no association between MTHFR C677T polymorphism, HPV infection and cervical intraepithelial neoplasia was found.

## Data Availability

The datasets used and/or analyzed during the current study are available from the corresponding author on reasonable request.

## Abbreviations

C: Cytosine
CIN: Cervical Intraepithelial Neoplasia
dNTP: Deoxyribonucleotide
HPV: Human Papillomavirus
hr-HPV: high-risk Huma Papillomavirus
HWE: Hardy-Weinberg Equilibrium
MTHFR: Methylenetetrahydrofolate reductase
PCR: Polymerase Chain Reaction
RFLP: Restriction Fragment Length Polymorphism
SCC: Squamous Cervical Carcinoma
SNP: Single Nucleotide Polymorphism
T: Thymine
